# Epidemiological, Clinical, and Therapeutic Characteristics of Idiopathic Granulomatous Mastitis Treated With Ultrasound-Guided Intralesional Steroid Injection

**DOI:** 10.7759/cureus.111373

**Published:** 2026-06-23

**Authors:** Larissa Bitencourt, Luiza Mascarenhas

**Affiliations:** 1 Breast Surgery, Hospital Santo Antonio, Salvador, BRA

**Keywords:** breast inflammation, conservative treatment, idiopathic granulomatous mastitis, intralesional steroid, triamcinolone acetonide, ultrasound-guided injection

## Abstract

Background: Idiopathic granulomatous mastitis is a rare benign chronic inflammatory breast disease with heterogeneous clinical and radiologic presentations and no standardized treatment. This study aimed to describe the epidemiological, clinical, radiologic, and therapeutic characteristics of patients with biopsy-proven idiopathic granulomatous mastitis treated with ultrasound-guided intralesional steroid injection.

Methods: We conducted a retrospective observational study at a breast department in Salvador, Brazil. Medical records from May 2023 to January 2024 were reviewed. Patients with histologically confirmed granulomatous mastitis, negative bacterial and fungal cultures, and treatment with ultrasound-guided intralesional triamcinolone acetonide were included. Clinical, radiologic, treatment, and response data were analyzed descriptively.

Results: Ten females were included. The mean age was 35.8 years, and all patients were premenopausal. Pain or tenderness with hyperemia was observed in nine patients (90%). All patients had skin involvement, and three (30%) had fistulas. The mean number of intralesional steroid injections was 3.3, and the mean follow-up was 5.7 months. Complete clinical response occurred in five patients (50%), partial response in three (30%), and no response in two (20%). Complete radiologic response occurred in four patients (40%). No patient required surgery. One patient developed skin retraction.

Conclusions: Ultrasound-guided intralesional triamcinolone injection appears to be a feasible conservative treatment option for idiopathic granulomatous mastitis, with clinical or radiologic response in most patients and limited adverse events. Larger prospective studies are needed to define optimal dosing, durability of response, recurrence rates, and long-term safety.

## Introduction

Idiopathic granulomatous mastitis is a rare, benign, chronic inflammatory breast disease of uncertain etiology that predominantly affects women of reproductive age [1]. Proposed associations include hormonal factors, particularly estrogen, progesterone, and prolactin, as well as obesity, genetic susceptibility, autoimmune disease, and diabetes. However, its underlying pathophysiology remains incompletely understood.

The clinical and radiologic presentation of idiopathic granulomatous mastitis is heterogeneous and may delay diagnostic suspicion. Patients may present with a palpable breast mass and inflammatory findings, including pain, warmth, erythema, and increased local temperature. Because these manifestations may overlap with inflammatory breast cancer and other causes of mastitis, histologic confirmation is required to establish the diagnosis and exclude malignant and infectious conditions.

Diagnosis is based on histologic evaluation of tissue obtained from the lesion. Core needle biopsy is highly accurate and commonly used, whereas surgical biopsy, either incisional or excisional, may be considered in selected cases [2,3]. Histologically, idiopathic granulomatous mastitis is characterized by lobulocentric noncaseating granulomatous inflammation with epithelioid histiocytes, in the absence of specific infectious agents, trauma, or foreign body reaction [3].

Management remains controversial because the disease is uncommon and no standardized treatment has been established. Therapeutic options range from observation and conservative medical therapy, including antibiotics, anti-inflammatory drugs, and systemic or topical corticosteroids, to surgical treatment, including lesion excision and, in selected cases, mastectomy [4].

Systemic corticosteroids are among the main conservative strategies and may be effective [5]. However, high doses and prolonged treatment may limit their use because of systemic adverse effects and potential impact on quality of life. Topical corticosteroids were introduced to reduce systemic exposure and increase local action, but their effectiveness may be limited in extensive or deep lesions and when the overlying skin is intact.

Intralesional corticosteroid injection has emerged as a conservative alternative that maintains local steroid delivery while reducing systemic exposure. This approach may be particularly useful for larger or deeper lesions and for cases in which the skin barrier is preserved [5]. The present study aimed to describe the epidemiological, clinical, radiologic, and therapeutic characteristics of patients with biopsy-proven idiopathic granulomatous mastitis treated with ultrasound-guided intralesional triamcinolone acetonide injection.

## Materials and methods

Study design and setting

This retrospective observational study was conducted at a breast department in Salvador, Bahia, Brazil. Medical records of patients treated between May 2023 and January 2024 were reviewed.

The extracted variables included sex, origin, age at presentation, need for hospitalization, occupation, lifestyle factors, comorbidities, prior treatments, reproductive history, clinical signs and symptoms, number of visits, number of intralesional applications, imaging findings, treatment response, and adverse events.

Race and ethnicity data were sought but were not systematically recorded in the medical records during the study period and therefore could not be reliably extracted for analysis.

Eligibility criteria

Patients were eligible if they had histologically confirmed granulomatous mastitis, negative bacterial and fungal cultures, and treatment with ultrasound-guided intralesional corticosteroid injection using triamcinolone acetonide.

Patients were excluded if they were pregnant or postpartum with lactational mastitis; had a diagnosis of mastitis other than granulomatous mastitis; received only oral corticosteroids, other therapies, or expectant management; did not have cultures performed; or had positive bacterial or fungal cultures.

Treatment protocol

The institutional protocol consisted of ultrasound-guided intralesional administration of up to 80 mg of triamcinolone acetonide (20 mg/mL) every three to four weeks. Triamcinolone was diluted in lidocaine without a vasoconstrictor at a 1:2 ratio.

When fluid collections were present, aspiration was performed before steroid injection. Care was taken to avoid injection too close to the skin or muscle and to avoid blood vessels.

Response assessment

Clinical and radiologic responses were assessed retrospectively from medical records and imaging reports. Clinical response was classified as complete response, partial response, or no response according to documented changes in symptoms and physical examination findings. Complete clinical response was defined as complete documented resolution of breast symptoms and inflammatory physical examination findings, including pain or tenderness, hyperemia, active wound, fistula, or palpable inflammatory abnormality. Partial clinical response was defined as documented improvement in symptoms or physical examination findings without complete resolution of inflammatory signs or residual palpable abnormality. No clinical response was defined as absence of documented clinical improvement, persistence of active inflammatory findings, or clinical worsening during follow-up.

Radiologic response was classified as complete response, partial response, or no response according to follow-up breast imaging documentation. Complete radiologic response was defined as complete disappearance of the active inflammatory lesion on follow-up imaging or residual findings interpreted as inactive scar tissue, without persistent collection, fistulous tract, or active inflammatory change. Partial radiologic response was defined as documented reduction in lesion size or extent, or partial resolution of collections, fistulous tracts, tubular extensions, skin thickening, or inflammatory parenchymal changes, with residual abnormality still present. No radiologic response was defined as absence of meaningful interval improvement, persistence of an active inflammatory lesion, persistent collection or fistulous tract, or radiologic worsening on follow-up imaging.

Statistical analysis

All statistical analyses were performed using IBM SPSS Statistics for Windows, version 29.0 (IBM Corp., Armonk, NY, USA). Continuous variables were summarized as mean (standard deviation) and/or median (interquartile range), as appropriate. Categorical variables were summarized as counts and percentages.

Given the very small sample size (n = 10), the statistical analysis was primarily descriptive. Exploratory bivariate cross-tabulations were performed to identify possible patterns associated with clinical and radiologic response. Pearson chi-square tests were reported only as exploratory analyses when cross-tabulation was feasible; however, because several expected cell counts were below accepted thresholds, these p-values were interpreted with considerable caution and were not considered confirmatory. No multivariable analysis was performed.

Ethics

The study protocol was approved by the institutional research ethics committee and registered under reference number 75506223.0.0000.0047 on November 30, 2023. The requirement for written informed consent was waived by the ethics committee because of the retrospective design of the study. Patient privacy and confidentiality were preserved throughout data collection, analysis, and reporting.

## Results

Demographic and baseline characteristics

From May 2023 to January 2024, 10 patients with biopsy-proven idiopathic granulomatous mastitis met the inclusion criteria. Baseline characteristics and selected risk factors are summarized in Table 1.

**Table 1:** Demographic and baseline characteristics of the patients with idiopathic granulomatous mastitis. BMI, body mass index; SD, standard deviation. Race and ethnicity data were not systematically recorded in the medical records during the study period and therefore could not be reliably extracted for analysis.

Characteristic	Overall (n = 10), n (%) or mean (SD)
Age at presentation, years, mean (SD)	35.8 (6.12)
BMI (kg/m²), mean (SD)	31.64 (6.85)
Origin, n (%)	
Salvador	6 (60%)
Other	4 (40%)
Marital status, n (%)	
Single	6 (60%)
Married	4 (40%)
Education level, n (%)	
Incomplete grade school	2 (20%)
Completed high school	6 (60%)
Bachelor’s degree	2 (20%)
Menarche, n (%)	
<12 years	2 (20%)
>=12 years	8 (80%)
Menstrual cycle, n (%)	
Normal cycle	6 (60%)
Abnormal cycle	2 (20%)
Continuous hormonal contraceptive	1 (10%)
Amenorrhea	1 (10%)
Menopausal status, n (%)	
Premenopausal	10 (100%)
Hormonal contraceptive use among ever-users, n (%)	
<5 years	4 (50%)
>=5 to <10 years	1 (12.5%)
>=10 years	3 (37.5%)
First live birth, n (%)	
<30 years	9 (90%)
>=30 years	1 (10%)
Breast-feeding, n (%)	
>=6 months	8 (80%)
No	2 (20%)
Family history of breast cancer (first-degree relative), n (%)	1 (10%)
Smoker in the last 5 years, n (%)	0
Alcohol consumption, n (%)	
Yes	4 (40%)
No	6 (60%)
Physical exercise, n (%)	
Yes	4 (40%)
No	6 (60%)
Healthy eating, n (%)	
Yes	4 (40%)
No	6 (60%)
Insomnia, n (%)	
Yes	6 (60%)
No	4 (40%)

All patients were women and were premenopausal at presentation. The mean age was 35.8 years (standard deviation (SD), 6.12; range, 28-45 years), and the mean body mass index was 31.64 kg/m² (SD, 6.85). Six patients (60%) were from Salvador, Bahia, Brazil, and six (60%) were single. High school completion was the most frequent education level, reported by six patients (60%).

Eight patients (80%) had menarche at 12 years of age or later, and six (60%) reported regular menstrual cycles. Among patients with previous hormonal contraceptive use, four (50%) had used hormonal contraception for less than five years. Nine patients (90%) had their first live birth before 30 years of age, and eight (80%) had breastfed for at least six months. One patient (10%) had a first-degree family history of breast cancer. No patient reported smoking in the preceding five years. Six patients (60%) reported no alcohol consumption, no regular physical exercise, unhealthy eating habits, and insomnia.

Clinical presentation

The most common presentation was pain or tenderness associated with hyperemia, observed in nine patients (90%). All patients had skin involvement, including a bulge in two patients (20%), a scar in two (20%), a fistula in three (30%), and a wound in two (20%). Abnormal palpation was documented in eight patients (80%); among these, four patients had dense areas, and four had nodules. No patient had axillary abnormalities or nipple discharge. Clinical characteristics are summarized in Table 2.

**Table 2:** Clinical characteristics of patients with idiopathic granulomatous mastitis. Percentages for dense area and nodule under abnormal palpation are calculated among patients with abnormal palpation (n = 8). Categories of skin involvement are reported as documented in the medical records.

Characteristic	Overall (n = 10), n (%)
Symptoms reported at presentation, n (%)	
Nodule	1 (10%)
Pain/tenderness plus hyperemia	9 (90%)
Skin involvement, n (%)	
Bulge	2 (20%)
Scar	2 (20%)
Fistula	3 (30%)
Wound	2 (20%)
Abnormal palpation, n (%)	8 (80%)
Dense area	4 (50%)
Nodule	4 (50%)
Axillary abnormality, n (%)	0
Nipple discharge, n (%)	0

Radiologic findings

Mammography was performed in five patients (50%). Among these, three patients (60%) had Breast Imaging Reporting and Data System (BI-RADS) category 2 findings, one (20%) had BI-RADS category 0 findings, and one (20%) had BI-RADS category 4 findings.

Breast ultrasonography was performed in all patients and was the primary imaging modality used for diagnosis and follow-up. BI-RADS 2 was observed in six patients (60%), BI-RADS 3 in one patient (10%), and BI-RADS 4 in three patients (30%). In addition to BI-RADS classification, the documented sonographic morphology included fluid collections in five patients (50%), parenchymal distortion in two patients (20%), and nodular findings in three patients (30%). These morphologic findings were extracted from the available retrospective imaging reports. No additional standardized sonographic descriptors, such as tubular extensions or skin thickening measurements, were consistently available in all reports.

One patient underwent breast magnetic resonance imaging, which showed BI-RADS 4 findings. Imaging modalities and BI-RADS distributions are summarized in Table 3.

**Table 3:** Radiologic findings of patients with idiopathic granulomatous mastitis. BI-RADS, Breast Imaging Reporting and Data System; MRI, magnetic resonance imaging. Percentages for imaging performance are calculated from the total cohort. Percentages for BI-RADS categories are calculated among patients who underwent the corresponding imaging modality.

Imaging modality and finding	Overall (N = 10), n/N (%)
Mammography performed	5/10 (50%)
BI-RADS 0	1/5 (20%)
BI-RADS 2	3/5 (60%)
BI-RADS 4	1/5 (20%)
Breast ultrasonography performed	10/10 (100%)
BI-RADS 2	6/10 (60%)
BI-RADS 3	1/10 (10%)
BI-RADS 4	3/10 (30%)
Breast MRI performed	1/10 (10%)
BI-RADS 4	1/1 (100%)

Treatment and response

Treatment characteristics are presented in Table 4.

**Table 4:** Treatment characteristics of patients with idiopathic granulomatous mastitis treated with ultrasound-guided intralesional steroid injection. SD, standard deviation. ^a^ Calculated among patients who achieved complete clinical response.

Characteristic	Overall (n = 10), n (%) or mean (SD)
Number of intralesional steroid injections, mean (SD)	3.3 (1.94)
Follow-up, months, mean (SD)	5.7 (2.31)
Time to complete clinical response, months, mean (SD)^a^	6.8 (1.64)
Topical triamcinolone acetonide, n (%)	
Yes	3 (30%)
No	7 (70%)
Therapies before intralesional applications, n (%)	
Oral corticosteroid	2 (20%)
Antibiotic	2 (20%)
Oral corticosteroid + antibiotic	4 (40%)
Oral corticosteroid + methotrexate	2 (20%)
Side effects, n (%)	
No	9 (90%)
Skin retraction	1 (10%)

The mean number of ultrasound-guided intralesional steroid injections was 3.3 (SD, 1.94; range, 1-6), and the mean follow-up was 5.7 months (SD, 2.31; range, 1-8 months). Three patients (30%) also received topical triamcinolone acetonide.

All patients had received treatment before the intralesional corticosteroid injection. Previous therapies included oral corticosteroids in two patients (20%), antibiotics in two (20%), oral corticosteroids combined with antibiotics in four (40%), and oral corticosteroids combined with methotrexate in two (20%). The interval between completion of prior systemic therapies and initiation of intralesional treatment was not consistently documented in the medical records and therefore could not be reliably analyzed. Consequently, the potential residual effects of previous systemic treatments on subsequent response could not be fully assessed.

Two patients who had previously received oral corticosteroids combined with methotrexate continued methotrexate during the intralesional injection protocol. Among these two patients, one achieved a partial clinical response, and one had no clinical response. Radiologic response in this subgroup was partial in one patient and absent in one patient. Because ongoing methotrexate may have influenced inflammatory disease activity, treatment outcomes in these patients should be interpreted as potentially confounded.

Among the three patients who received adjunctive topical triamcinolone acetonide, complete clinical response occurred in one patient and partial clinical response occurred in two patients; no patient in this subgroup was classified as having no clinical response. Radiologic response in this subgroup was complete in one patient and partial in two patients, with no cases classified as no radiologic response. Because topical corticosteroid therapy was used concomitantly with the intralesional protocol, these responses should be interpreted as potentially influenced by adjunctive topical treatment.

**Figure 1:**
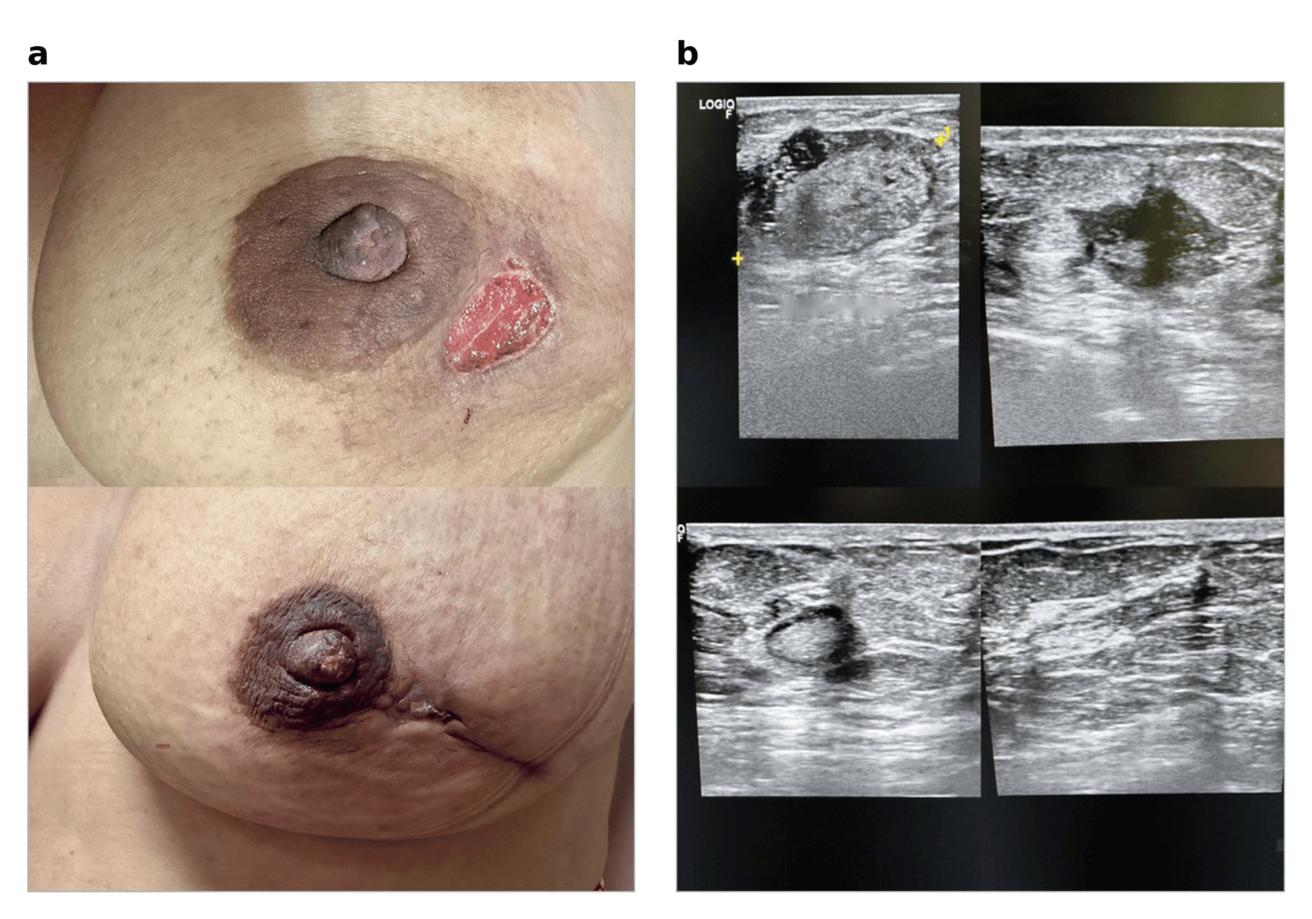
Representative clinical and ultrasonographic findings related to intralesional steroid treatment. (a) Clinical appearance before and after intralesional steroid treatment. The upper clinical photograph shows a wound at presentation; the lower clinical photograph, obtained six months later, shows scar formation and skin retraction. (b) Breast ultrasonography images before and after ultrasound-guided intralesional corticosteroid injection, showing resolution of the baseline sonographic abnormality and complete radiologic response on follow-up imaging.

No patient required surgical treatment. No allergic reaction to topical or intralesional triamcinolone acetonide was observed. One patient (10%) developed skin retraction as a local adverse event. Representative clinical and ultrasonographic findings before and after treatment are shown in Figure 1.

**Figure 2:**
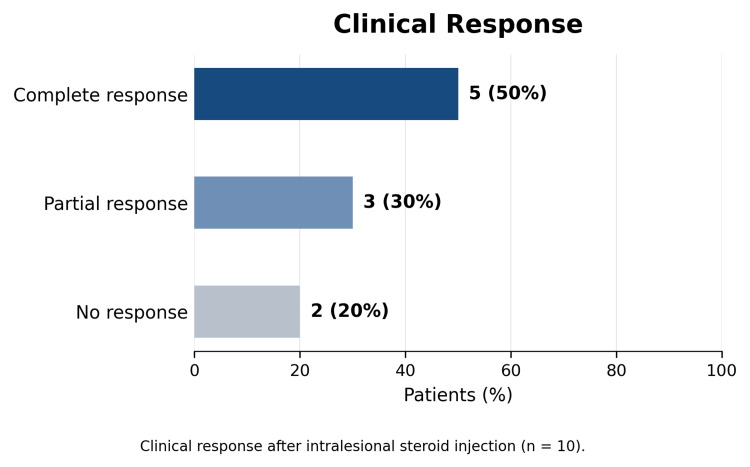
Clinical response after ultrasound-guided intralesional steroid injection. Complete clinical response was observed in five patients (50%), partial clinical response in three patients (30%), and no clinical response in two patients (20%). Data are presented as the number and percentage of patients in the total cohort (n = 10).

Complete clinical response was observed in five patients (50%), partial clinical response in three (30%), and no clinical response in two (20%) (Figure 2).

Among patients who achieved complete clinical response, the mean time to complete response was 6.8 months (SD, 1.64; range, 4-8 months). In exploratory bivariate analysis, age younger than 30 years showed a nominal association with complete clinical response (p = 0.035). No comparable association was observed among the other evaluated variables. This finding should be interpreted with considerable caution because of the very small sample size, sparse expected cell counts, and multiple exploratory comparisons, and should be regarded as hypothesis-generating rather than confirmatory. The exploratory statistical analysis results are presented in Table 5.

**Table 5:** Exploratory statistical analysis of factors associated with complete clinical response. BI-RADS, Breast Imaging Reporting and Data System; MRI, magnetic resonance imaging. Given the small sample size, all inferential analyses were considered exploratory. Pearson chi-square p-values were reported only when cross-tabulation was feasible and should be interpreted cautiously because expected cell counts were less than five in several comparisons. Variables with constant values, including sex, smoking status, hormone replacement therapy, and adverse reactions to intralesional applications, were not included because chi-square statistics could not be computed.

Variable evaluated	Outcome evaluated	Pearson chi-square p-value
Age younger than 30 years	Clinical response	p = 0.035
Origin	Clinical response	p = 0.143
Marital status	Clinical response	p = 0.329
Education level	Clinical response	p = 0.377
Body mass index	Clinical response	p = 0.333
Initial clinical presentation	Clinical response	p = 0.574
Menarche	Clinical response	p = 0.392
Breastfeeding	Clinical response	p = 0.659
Age at first live birth	Clinical response	p = 0.108
First-degree family history of breast or ovarian cancer	Clinical response	p = 0.574
Alcohol consumption	Clinical response	p = 0.933
Previous hormonal contraceptive use	Clinical response	p = 0.392
Regular physical activity	Clinical response	p = 0.143
Healthy eating habits	Clinical response	p = 0.933
Breast ultrasonography BI-RADS category	Clinical response	p = 0.084
Mammography BI-RADS category	Clinical response	p = 0.177
Number of intralesional steroid injections	Clinical response	p = 0.672
Inspection abnormality	Clinical response	p = 0.574
Palpation abnormality	Clinical response	p = 0.258
Type of inspection finding	Clinical response	p = 0.831
Type of palpation finding	Clinical response	p = 0.465
Menstrual pattern	Clinical response	p = 0.226
Sleep disturbance	Clinical response	p = 0.233
Previous therapy type	Clinical response	p = 0.264
Breast MRI BI-RADS category	Clinical response	p = 0.274

**Figure 3:**
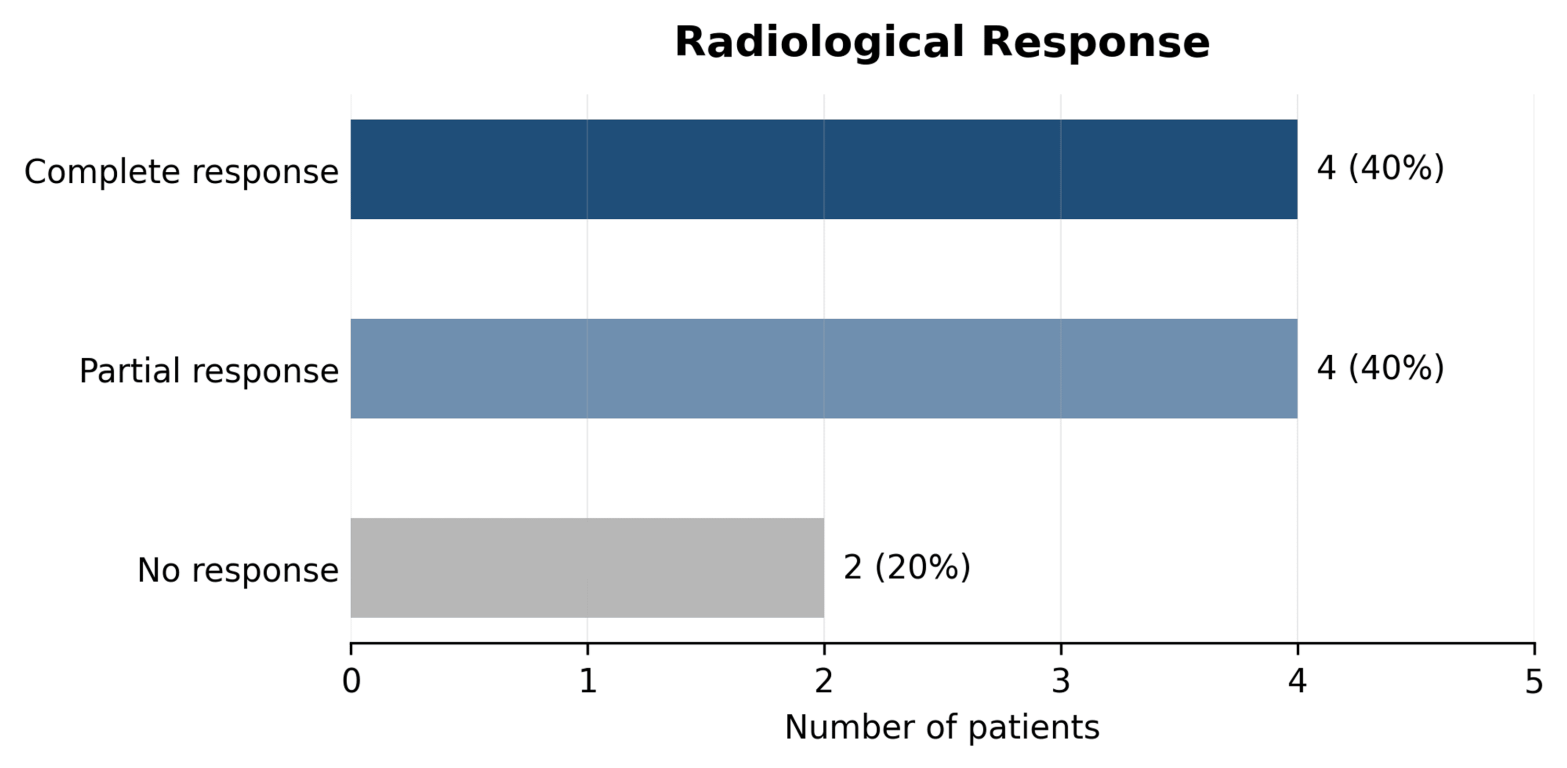
Radiologic response after ultrasound-guided intralesional corticosteroid treatment. Complete radiologic response was observed in four patients (40%), partial radiologic response in four patients (40%), and no radiologic response in two patients (20%). Data are presented as the number and percentage of patients in the total cohort (n = 10).

Complete radiologic response was observed in four patients (40%), partial radiologic response in four (40%), and no radiologic response in two (20%) (Figure 3). Baseline lesion dimensions and follow-up measurements were reviewed when available; however, paired measurements were not consistently documented across baseline and follow-up imaging reports and were not sufficiently standardized to allow a reliable quantitative comparison of lesion size for the full cohort. Therefore, radiologic response was assessed qualitatively based on the documented resolution, reduction, persistence, or progression of active inflammatory findings on follow-up imaging.

## Discussion

Idiopathic granulomatous mastitis is an uncommon benign inflammatory breast disease with uncertain pathophysiology, heterogeneous clinical presentation, and no universally accepted therapeutic standard. In this retrospective series, ultrasound-guided intralesional triamcinolone injection was associated with clinical or radiologic response in most patients, no requirement for surgical treatment, and only one documented local adverse event.

Several mechanisms have been proposed for the development of idiopathic granulomatous mastitis. One hypothesis suggests that factors promoting the accumulation of secretion within mammary ducts may contribute to disease onset. Increased ductal permeability, residual milk after lactation, and subsequent ductal obstruction may lead to ductal rupture, epithelial injury, and extravasation of secretion into the breast parenchyma, thereby triggering humoral and cellular immune responses [6]. In this context, factors associated with ductal obstruction, ductal dilation, and accumulation of intraductal secretion may contribute to local inflammation.

Hormonal factors have also been implicated in the pathogenesis of idiopathic granulomatous mastitis. Changes in estrogen, progesterone, and prolactin levels, including those associated with hormonal contraceptive use, may stimulate ductal epithelial proliferation and contribute to ductal obstruction and inflammation. This hypothesis may help explain why idiopathic granulomatous mastitis is most frequently reported among women of reproductive age, with a mean age at onset between 32 and 35 years in previous series [1]. The demographic profile of the present cohort, composed exclusively of premenopausal women with a mean age of 35.8 years, is consistent with this pattern.

Psychological and mood disorders, including anxiety and depression, have also been discussed in relation to idiopathic granulomatous mastitis. Increased levels of proinflammatory cytokines, such as tumor necrosis factor alpha and interleukin-6, have been described in patients with emotional disorders and may contribute to inflammatory responses. In addition, some medications used to treat these conditions may be associated with hyperprolactinemia. Hyperprolactinemia has been implicated in idiopathic granulomatous mastitis because it may stimulate secretion accumulation, ductal dilation, ductal obstruction, and local inflammation [7].

Although these associations do not establish causality, behavioral measures such as regular physical activity, healthy dietary habits, and preservation of the sleep-wake cycle may be encouraged during follow-up as part of comprehensive care. In the present study, most patients had obesity, did not report regular physical exercise or healthy eating habits, and reported insomnia. These variables were not tested as independent predictors in this small series and should therefore be interpreted descriptively. Smoking has also been described as a possible associated factor in previous studies [7], but no patient in this cohort reported smoking in the preceding five years.

The clinical presentation in this series differed from some previously published data in which a palpable mass was the main symptom at first consultation [1]. In the present cohort, the most frequent presentation was pain or tenderness associated with hyperemia. Local inflammatory signs, including pain, warmth, erythema, and increased local temperature, are relevant features of idiopathic granulomatous mastitis and may overlap with other inflammatory breast conditions, including infectious mastitis and inflammatory breast carcinoma. The frequency of fistulas, which characterizes complicated disease, was similar to that reported by Ertürk et al. [4] and higher than that reported by Barreto et al. [1].

Breast ultrasonography was the main imaging method used for diagnosis and follow-up. This is consistent with the age profile of affected patients and with the accessibility of ultrasonography in clinical practice. Similar patterns have been reported in previous studies [1,2]. Most ultrasonographic findings in this series were classified as benign or probably benign, although BI-RADS 4 findings were also observed. Breast magnetic resonance imaging was performed in only one patient, consistent with the limited use of this modality reported in prior series [1,2].

There is no consensus regarding optimal treatment for idiopathic granulomatous mastitis. Management may range from observation to medical treatment with antibiotics, corticosteroids, or immunosuppressive agents and, in selected cases, surgery. Therapeutic strategies have generally been developed according to the proposed etiologic pathways of the disease, including infectious, autoimmune, and hormonal mechanisms.

The use of antibiotics has been supported by reports of *Corynebacterium* growth in tissue samples from patients with idiopathic granulomatous mastitis. The effectiveness of corticosteroids and immunosuppressive agents supports the hypothesis that idiopathic granulomatous mastitis may behave as an immune-mediated disease [7]. According to this model, triggering factors, such as hyperprolactinemia or accumulation of intraductal secretion, may damage the ductal epithelium and stimulate a T cell-mediated autoimmune inflammatory response, leading to the formation of noncaseating granulomas. Corticosteroids and immunosuppressive agents may control clinical manifestations by inhibiting this exacerbated immune response.

Therefore, it is common for patients to receive empirical treatment before the diagnosis is established, as well as additional therapeutic interventions after histologic confirmation. Antibiotics and systemic corticosteroids, alone or in combination, are among the most frequently described treatments in the literature. This pattern was also observed in the present study because all patients had received previous therapies before initiation of the intralesional corticosteroid protocol.

Intralesional corticosteroid therapy has been compared with observation, oral corticosteroids, and surgical treatment. Compared with observation, intralesional corticosteroid injection has been associated with shorter disease duration, faster resolution, lower recurrence rates, and fewer adverse events [8]. Compared with systemic corticosteroids, intralesional treatment appears to have similar effectiveness, with studies reporting faster response, shorter follow-up, lower recurrence and surgery rates, and fewer side effects [2].

Compared with surgical treatment, intralesional corticosteroid therapy has been associated with better cosmetic outcomes, lower recurrence, lower cost, and greater effectiveness in pain control [4]. In the present study, no patient required surgery. The mean follow-up was similar to that reported in the current literature [8], and the mean number of injections was 3.3. In the study by Ertürk et al., the mean number of injections was three among patients with fistulas and two among patients without fistulas [4]. The low frequency of local and systemic adverse events represents a potential advantage of intralesional therapy, particularly when compared with oral corticosteroids, and this was also observed in our series [2].

Overall, eight of 10 patients showed some clinical response to the proposed treatment. Complete clinical response was observed in five patients, and complete radiologic response was observed in four patients. Among patients who achieved complete clinical response, the mean time to response was 6.8 months. These findings are consistent with response rates reported in the literature [2,4]. Age younger than 30 years showed a nominal exploratory association with complete clinical response in this cohort. This finding should not be interpreted as a definitive predictor of response because the analysis was limited by the very small sample size, sparse expected cell counts, and multiple exploratory comparisons. Therefore, this result should be considered hypothesis-generating only and requires confirmation in larger prospective cohorts.

Study limitations

This study has limitations. Idiopathic granulomatous mastitis is a rare condition, and the number of patients included in this retrospective cohort was small. The retrospective design limits control over data completeness, selection bias, timing of follow-up assessments, and standardization of clinical and imaging documentation. In addition, the absence of a comparison or control group prevents conclusions regarding comparative effectiveness or superiority over other treatment strategies.

Clinical and radiologic responses were assessed retrospectively from medical records and imaging reports. Although explicit response definitions were applied in this study, the original clinical and imaging evaluations were not performed using a prospective standardized response protocol. Baseline lesion dimensions and follow-up measurements were reviewed when available; however, paired measurements were not consistently documented across baseline and follow-up imaging reports and were not sufficiently standardized to allow reliable quantitative comparison of lesion size for the full cohort. As a result, radiologic response was assessed qualitatively based on documented resolution, reduction, persistence, or progression of active inflammatory findings on follow-up imaging.

Ultrasonography was the primary imaging modality used for diagnosis and follow-up. However, the level of sonographic morphologic detail available in the retrospective imaging reports varied among patients. Therefore, although collections, parenchymal distortion, and nodular findings could be described, other potentially relevant descriptors, such as tubular extensions, fistulous tracts, skin thickening measurements, and detailed assessment of residual scar tissue, were not consistently available across all reports.

Prior and concomitant therapies may have influenced treatment outcomes. The interval between prior systemic therapies and initiation of intralesional treatment was not consistently documented in the medical records and therefore could not be reliably analyzed. In addition, two patients continued methotrexate during the intralesional injection protocol, and three patients received adjunctive topical triamcinolone acetonide. These factors may have confounded treatment response and limited attribution of the observed outcomes exclusively to intralesional corticosteroid injection.

The statistical analyses were exploratory only. Because of the very small sample size, sparse expected cell counts, and multiple exploratory comparisons, the reported p-values should not be considered confirmatory. No multivariable analysis was performed. Therefore, the nominal association observed between age younger than 30 years and complete clinical response should be interpreted as hypothesis-generating only and requires confirmation in larger prospective cohorts.

Finally, the follow-up period was relatively short, which limits evaluation of long-term recurrence, durability of response, and delayed adverse events. Prospective studies with larger sample sizes, standardized clinical and radiologic response criteria, objective imaging measurements, detailed sonographic descriptors, predefined statistical methods, and longer follow-up are needed to better define predictors of response, recurrence rates, safety, and the optimal number and interval of ultrasound-guided intralesional corticosteroid injections.

## Conclusions

In this small retrospective cohort, ultrasound-guided intralesional triamcinolone injection was associated with clinical or radiologic improvement in most patients with biopsy-proven idiopathic granulomatous mastitis. No patient required surgery during follow-up, and only one local adverse event, i.e., skin retraction, was documented. These findings suggest that intralesional corticosteroid injection may be a feasible conservative treatment option for selected patients with idiopathic granulomatous mastitis.

However, the results should be interpreted cautiously because of the retrospective design, small sample size, absence of a control group, lack of standardized quantitative imaging measurements, and potential confounding from prior or concomitant therapies. Prospective studies with larger sample sizes, standardized clinical and radiologic response criteria, objective imaging measurements, and longer follow-up are needed to define optimal dosing, durability of response, recurrence rates, and long-term safety.
